# An Insight into the Machine-Learning-Based Fileless Malware Detection

**DOI:** 10.3390/s23020612

**Published:** 2023-01-05

**Authors:** Osama Khalid, Subhan Ullah, Tahir Ahmad, Saqib Saeed, Dina A. Alabbad, Mudassar Aslam, Attaullah Buriro, Rizwan Ahmad

**Affiliations:** 1 FAST School of Computing, National University of Computer and Emerging Sciences (NUCES-FAST), Islamabad 44000, Pakistan; 2 Center for Cybersecurity, Brunno Kessler Foundation, 38123 Trento, Italy; 3 SAUDI ARAMCO Cybersecurity Chair, Department of Computer Information Systems, College of Computer Science and Information Technology, Imam Abdulrahman Bin Faisal University, P.O. Box 1982, Dammam 31441, Saudi Arabia; 4 SAUDI ARAMCO Cybersecurity Chair, Department of Computer Engineering, College of Computer Science and Information Technology, Imam Abdulrahman Bin Faisal University, P.O. Box 1982, Dammam 31441, Saudi Arabia; 5 Faculty of Computer Science, Free University Bozen-Bolzano, 39100 Bolzano, Italy; 6 School of Electrical Engineering and Computer Science, National University of Sciences and Technology (NUST), Islamabad 44000, Pakistan

**Keywords:** malware, filelss malware, volatility, cybercrimes, machine learning, memory forensics

## Abstract

In recent years, massive development in the malware industry changed the entire landscape for malware development. Therefore, cybercriminals became more sophisticated by advancing their development techniques from file-based to fileless malware. As file-based malware depends on files to spread itself, on the other hand, fileless malware does not require a traditional file system and uses benign processes to carry out its malicious intent. Therefore, it evades conventional detection techniques and remains stealthy. This paper briefly explains fileless malware, its life cycle, and its infection chain. Moreover, it proposes a detection technique based on feature analysis using machine learning for fileless malware detection. The virtual machine acquired the memory dumps upon executing the malicious and non-malicious samples. Then the necessary features are extracted using the Volatility memory forensics tool, which is then analyzed using machine learning classification algorithms. After that, the best algorithm is selected based on the k-fold cross-validation score. Experimental evaluation has shown that Random Forest outperforms other machine learning classifiers (Decision Tree, Support Vector Machine, Logistic Regression, K-Nearest Neighbor, XGBoost, and Gradient Boosting). It achieved an overall accuracy of 93.33% with a True Positive Rate (TPR) of 87.5% at zeroFalse Positive Rate (FPR) for fileless malware collected from five widely used datasets (VirusShare, AnyRun, PolySwarm, HatchingTriage, and JoESadbox).

## 1. Introduction

Malware—a computer program that compromises a target system by infecting the other programs installed on that system [1], is a significant threat to the security of individuals and organizations [1]. Malware could be used to steal sensitive data, disrupt operations, and cause damage to systems. In most cases, malicious programs are created to make illegal money by asking for ransomware in exchange for access to the user’s infected machine [2,3]. Cybercriminals use various techniques to create and distribute malware, including embedding it in legitimate software, disguising it as a legitimate application, or using phishing attacks to trick users into installing it.

Fileless malware is a type of malware that infects the computer system through its vulnerabilities and the legitimate programs installed. As the name describes, it does not fully rely on files. It targets the main memory of the computer system instead of the hard disk. Fileless malware is a serious threat to cybersecurity because it can evade traditional detection methods that rely on identifying malicious files on the hard disk. It can also be difficult to remove because it does not leave any trace of itself on the hard disk [4]. There are several ways for fileless malware to infect a computer system: One common method is using malicious scripts embedded in legitimate files or programs. The script is executed when the user opens the file or program and infects the system. Fileless malware can also be delivered through phishing attacks, where the user is tricked into clicking on a malicious link or downloading a malicious file. This malware became extremely popular in 2017 as an exponential surge of 29% in the attacks was observed [5]. To protect against fileless malware, it is important to practice safe browsing habits, such as avoiding clicking on suspicious links or downloading unknown files. It is also important to keep software and operating systems up-to-date and to use good quality antivirus software capable of detecting fileless malware. The notion of good quality means the installed antivirus should not be signature-dependent and heuristic-based detection [6]. Consequently, existing state-of-the-art techniques have failed to detect fileless malware because of its stealth capability [7]. It is also important to have a robust network and device configuration in place to prevent unauthorized access and monitor for any suspicious activity.

Machine learning, including deep learning, has indeed been very successful in various domains, including fileless malware detection [7,8,9,10,11]. These models can generalize well on unseen testing samples, and they are accurate in detecting malware. However, it is important to consider the performance measurement of these models in terms of training and decision time, their requirement of an increased number of training samples, and their vulnerability to adversarial attacks.

In this paper, we propose to use memory forensic techniques to extract the representative features of the fileless malware from the system’s main memory and use machine learning for prediction. The use of memory forensic techniques and machine learning in detecting fileless malware is a promising approach because it allows the detection of malware that may not leave any trace on the hard disk. Analyzing the features of fileless malware in the main memory makes it possible to identify patterns and characteristics that could be used to train machine-learning algorithms to detect these threats. However, it is notable that this approach may not be foolproof and may require ongoing updates and adjustments to the machine learning model to stay effective against new and evolving fileless malware threats. It is also important to have robust cybersecurity measures in place to prevent the initial infection of fileless malware, as well as to have a plan in place for responding to and mitigating the effects of a fileless malware attack which is out of the scope of this paper. The malware and non-malware (benign) samples are executed in the virtual machine individually, followed by creating a memory dump from the virtual machine. We used Volatility—a memory forensic tool [12]—to extract the features from memory dumps using different volatility plugins. The extracted features are stored as datasets for validation by machine learning classifiers. The main contributions of this work can be summarized as follows:

- The proposal of using memory forensic techniques in conjunction with advanced state-of-the-art machine-learning algorithms to detect fileless malware.
- The creation of a dataset (though smaller but worth it) using memory forensic techniques.
- Preliminary results on the created dataset using state-of-the-art machine learning algorithms.

The remainder of the paper is organized as follows: Section 2 discusses the related work, and Section 3 describes the types and working of malware. Section 4 presents steps we adapted for malware detection based on machine learning classifiers. Section 5 evaluates the approach experimentally, and Section 6 concludes and discusses future work.

## 2. Related Work

Currently, malware is using sophisticated approaches for cyber attacks and advances its attacking techniques from file-based to fileless attacks to bypass the existing solutions for malware detection [13]. These existing solutions [14,15] can easily detect file-based malware attacks on windows [16], Android [17,18], and IoT devices [19], but fail to detect the fileless malware. This section presents the literature review, and comparative analysis of machine learning approaches limited to fileless malware. Lee et al. [8] thoroughly analyzed the attack techniques of ten previously known fileless malware collected from public websites (e.g., Hybrid-analysis and GitHub) and proposed a classification method for them based on their attack technique and characteristics. They used Cuckoo Sandbox to analyze fileless malware and mapped them on MITRE ATT&CK kill-chain [20]. They classified the fileless malware into three categories (attack type, evasion, and collection) based on their results from Cuckoo Sandbox and improved the response time to fileless attacks. Sanjay et al. [9] also discussed the technical details of fileless malware attacks with their detection and mitigation techniques using heuristic-based malware analysis and sandboxing. They categorized fileless malware into RAM-resident fileless malware and script-based fileless malware. Then they described the evasion techniques used by the fileless malware that is tricking the victim into downloading the malicious document that contains a malicious script in the form of a macro. As the victim opens the malicious file, the script injected in the file executes and inserts the malicious code into the memory.

Afreen et al. [7] also studied different types of fileless malware and analyzed their infection and attack techniques along with their detection techniques. They defined various execution techniques of fileless malware on the target systems, which include .NET frameworks, Windows Management Instrumentation, and Power-Shell. They also discussed different fileless malware attacks that performed an attack on the target system and gained persistence to the system. They further clarified that improving the current analysis behavior can improve the fileless malware detection technique. Fileless malware not only infects the system through the infected file but can also infect the system through the browser. Saad et al. [10] used the features of JavaScript and HTML5 and a JavaScript-based fileless malware that targets the browser to infect the computer system by using the features such as WebSockets, Web Workers, and Service Workers of HTML5 and JavaScript. They tested their malware on different static and dynamic detection tools, and none of them detected their malware. They showed that the attackers could exploit these features to insert fileless malware in the browser and maintain its persistence by using WebSocket and Service Worker, respectively.

Fileless malware can also infect Linux-based IoT devices. Dang et al. [21] developed and deployed hardware and software-based honeypots in multiple public clouds. The honeypots capture fileless and IoT-based malware, analyze their behavior, and profile their characteristics. They observed 264 million malicious connections to their honeypots in one year, among which 1.5 million were fileless attacks. They identified the fileless attacks by thoroughly correlating the disclosed shell commands, monitoring file system modifications, recording data-flow traffic, and examining third-party internet reports. They identified the attacks of fileless malware and characterized them into eight types that include: occupying end systems by altering the device password, damaging the system data by removing or altering configuration files and programs, preventing system monitoring/auditing services, retrieving system information, stealing valuable data, launching network attacks, and launching attacks using no shell commands (e.g., SSH tunneling attacks). Malware can also infect or take control of a victim’s machine to mine cryptocurrencies by using the system resources without their knowledge. Such type of malware is called Cryptojacking or Cryptocurrency mining malware [22,23]. Varlioglu et al. [24] reviewed fileless Cryptojacking malware and studied different types of Cryptojacking malware, which include in-browser Cryptojacking, in-host Cryptojacking, and fileless Cryptojacking. Moreover, they proposed a digital forensics threat-hunting-oriented technique that can detect Cryptojacking fileless malware.

Borana et al. [11] briefly studied fileless malware and its life cycle and then proposed a detection tool for fileless malware. Their detection tool consists of different modules that can collect information about a running process and present that information to a system admin for decision. The collected information may include information about the running processes and their hierarchy (e.g., a list of child and parent processes). Then, they list the process with the priority based on the information of DLLs, network protocols, network connection state, local and foreign addresses, name and ID of a process, and port numbers. They created the process dump and uploaded it to the online scanner “Virus Total” and generated a report after scanning was complete. They triggered a warning to the system administrator for the desired action if the online scanner found something malicious.

Tancio [25] studied different variations of fileless malware, including code injection, script-based attacks, living off the land, and fileless persistence. In addition, they proposed the fileless malware detection technique with the help of memory forensics using the Volatility tool (Volatility is a command-line tool to extract the important information from the memory image that is helpful for the detection of malware [12]). Their technique is based on manual forensics analysis of the memory for the detection of fileless malware, which is a high time, effort, and resource-consuming task. Tarek et al. [26] defined rules called dynamic signatures to monitor the binaries of fileless malware and identify their malicious behavior. They discussed the detection techniques of antiviruses and the evasion techniques that hide malware from detection. Most antiviruses use signature-based detection techniques, which maintain the signatures of malware in the viral definition database of the antivirus programs. Antivirus programs use these signatures to scan for particular malware. Dynamic signature detection cannot be bypassed by malware using the above evasion techniques. They extracted the dynamic behavior of malware with the help of Microsoft Tool “Detours” and API hooking. Once they create the process, it will trigger the tool “Detours”, which injects the DLL created by the authors into the process to perform hooking. They collect DLL logs, API calls and arguments and send them back to the behavior analyzer that compares that extracted behavior with the signatures stored in the database and performs the desired action. For experimental purposes, they wrote three signatures: AMSI Bypass Detection, Lsass.exe process dump detection, and process hollowing detection. They can detect a malicious process created by fileless malware using these signatures.

Bucevschi et al. [27] presented an anomaly detection method for the command line arguments to extract the features that build a machine learning model for the detection of fileless malware using a perceptron algorithm called OSC. The perceptron algorithm ensures that all the correct classifications of benign samples perform extra training to minimize the number of falsely classified entries. They built a dataset for testing and training purposes that contains 500,551 command lines, PowerShell scripts, Windows Management instrumentation scripts, bash scripts, etc., provided by Bit-defender Cyber Threat Intelligence (CTI) lab and Virus Total Intelligence (VTI). They decided to use the ratio of five clean commands against malicious ones to reduce the rate of false positives. They further avoided false-positive situations when they extracted specialized features for malicious commands and cleaned the command lines with the extraction of features available in both categories to observe the malicious commands, anomalies, and obfuscation methods.

Table 1 shows the summary of different approaches for the detection of fileless malware. It is important for any tool that aims to detect and protect against malicious behavior to have a clear and understandable way of informing the user about any potential threats. Simply providing a warning to the user may not be enough, especially if the user lacks a technical background and does not know how to respond to the warning properly. One potential improvement to the approach proposed by Borana et al. [11] could be to provide more detailed information to the user about the detected threat, such as the specific process or DLL that triggered the warning and recommendations on how to address the issue. It could also be helpful to have an option for the tool to automatically take action to mitigate the threat, such as blocking the process or quarantining the file, with the user’s permission. It is also important to consider the potential risks and limitations of the tool, such as the possibility of false positives or the need for the user to consent before any action is taken. It may be helpful to have additional safeguards to ensure that the tool is used responsibly and does not cause unintended consequences. The proposed approach uses machine learning techniques with memory forensics and explored 33 different features of in-memory and browser-based fileless attacks. These fileless malware exploit PowerShell, WMI, Macros, and VB scripts for attacks. The proposed approach built their dataset for the fileless malware downloaded from virus total, AnyRun, and PolySwarm websites. Comparing the proposed work with the state-of-the-art provides more accurate classification and detection results of fileless malware.

## 3. Background

This section briefly discusses file-based and fileless malware’s infection chain and the working of malware.

### 3.1. File-Based Malware Infection Chain

**Figure 1:**
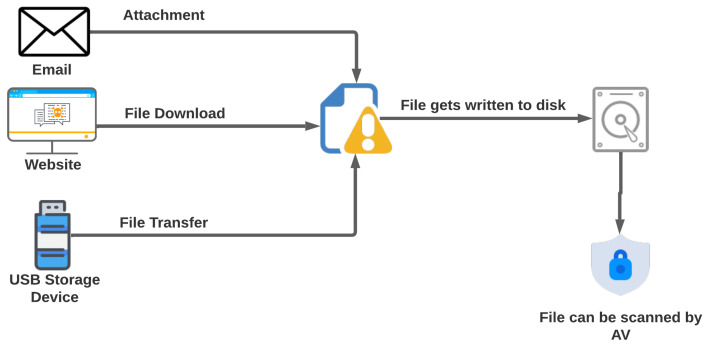
File-based malware infection chain (how malware infects the victim machine).

Figure 1 illustrates the infection chain of file-based malware. File-based malware is a type of malware that is transmitted through files, such as executables, documents, or scripts. These types of malware can infect a user’s system by being downloaded from a malicious website, received as an attachment in an email, or transferred through an infected flash drive. Once the infected file is received, it is written to the hard disk of the user’s system. If the user has an antivirus program installed, the file may be scanned by the antivirus program. If the antivirus program detects the file as malicious (malicious), it may remove or quarantine that file to prevent it from execution. If the antivirus program does not detect the file as malicious (which means it is benign), the user is allowed to execute it [13], which can lead to malware infecting the system. It is important for users to be cautious when downloading files from unknown sources and to keep their antivirus programs up to date to protect against file-based malware.

#### Life Cycle of File-Based Malware

**Figure 2:**
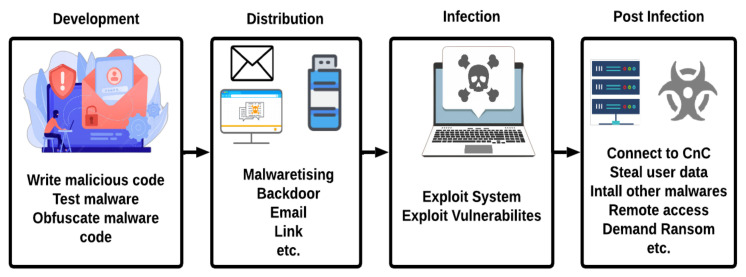
Life cycle of file-based malware.

Figure 2 shows the life cycle of file-based malware. It comprises the following four phases:

- **Development phase:**

The development phase of malware creation is the first step in the malware life cycle. During this phase, the malware author creates the code for the malware to cause damage to a victim’s computer or steal data from it. This phase is similar to the development phase of typical software, in which the code is written and tested to ensure it functions properly. During the development phase, the malware author may use various obfuscation techniques to avoid detection by antivirus programs [29]. These techniques include embedding, code appending, code surrounding, encryption, polymorphism, metamorphism, stealth, and packing. Obfuscation techniques make the malware more difficult to detect and analyze, making it harder for antivirus programs to identify and protect against it. Once the malware has been developed and tested, it can be distributed to potential victims. This can be achieved through various means, such as email attachments, malicious websites, or infected flash drives. Thus, it becomes important for users to be cautious when downloading files from unknown sources and to keep their antivirus programs up to date to protect against malware.

- **Distribution phase:**

The distribution stage of the malware life cycle is the phase in which the attacker attempts to deliver the malware to the victim’s machine. Various mechanisms can be used to accomplish this, such as social engineering, email attachments, exploit kits, and malvertizing [29]. Social engineering involves using psychological manipulation to convince the victim to take a certain action, such as clicking on a link or downloading an attachment. Attackers may use phishing emails or malicious websites to trick the victim into thinking they are interacting with a legitimate source. Email attachments can also be used to deliver malware. The attacker may send an email with a seemingly harmless attachment, but when the victim downloads and opens the attachment, the malware is executed on their machine. Exploit kits are tools used to exploit vulnerabilities in a victim’s system and install malware. They are often used to deliver malware through malicious websites or email attachments. Malvertizing is online advertising that uses malicious or infected ads to deliver malware to the victim’s machine. The malware is executed on their machine when the victim clicks on the ad.

- **Infection phase:**

During the infection stage of the malware life cycle [29], the goal of the attacker is to infect the victim’s system with the malware successfully. To achieve this, the attacker may use the malware to exploit vulnerabilities in the system. The attacker may also try to remain stealthy so that the malware is not detected by the victim’s antivirus or anti-malware programs. Several techniques help malware evade detection and infect the systems, namely, rootkits, hiding in legitimate processes, and using encryption.

- **Post-Infection phase:**

After successfully infecting a victim’s system, the malware will typically perform the actions it was designed to do. This can include a variety of malicious actions. Firstly, the malware may try to establish a connection with the command and control (CnC) server to receive further commands from the attacker. Secondly, the malware may try to steal sensitive data from the victim’s system, such as passwords, financial information, or personal documents. These data may be uploaded to the CnC server for the attacker to use. Thirdly, some types of malware, known as ransomware, are designed to encrypt the victim’s data and demand a ransom in exchange for the decryption key. Finally, the malware may give the attacker remote access to the victim’s system, allowing them to control the system remotely [29].

### 3.2. Fileless Malware Infection Chain

**Figure 3:**
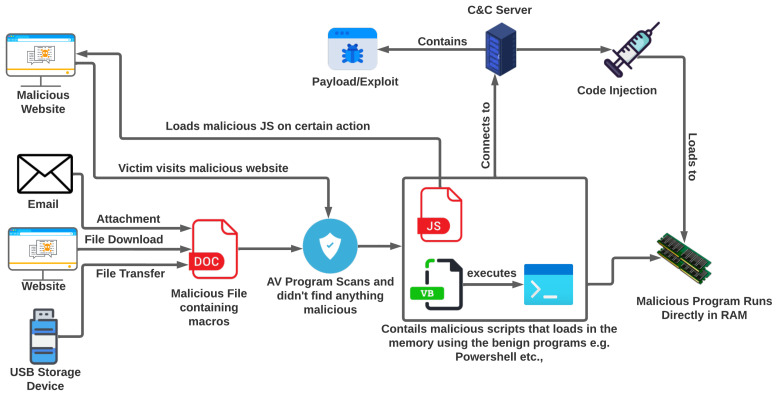
Fileless malware infection chain.

Figure 3 shows the infection chain of fileless malware. There are mainly two infection scenarios.

- **Infection Scenario 1:** Fileless malware is a type of malware that does not rely on traditional infection methods, such as downloading and executing a file on the victim’s system. Instead, it uses legitimate tools, such as PowerShell, to execute its code directly in the system’s memory. The initial steps of infection for fileless malware are similar to those of file-based malware. The victim may be tricked into downloading a file containing a malicious macro or script stored on the hard disk. However, when this file is executed, it uses PowerShell or another legitimate tool to execute its code in the system’s memory. Once the code is executing in the memory, the malware may trigger PowerShell to connect to a malicious Command and Control (CnC) server and download the final payload of the malware. This payload is then run directly in the system’s RAM without being stored on the hard disk [13]. Because fileless malware does not leave any trace on the hard disk, it can be more difficult to detect and remove than traditional file-based malware. It is important for users to be cautious when interacting with unknown links or downloading attachments and to use antivirus and antimalware software to protect against fileless malware.
- **Infection Scenario 2:** The user can be infected by visiting a malicious website containing malicious JavaScript triggered by some particular action. At this point, if an antivirus scans the website, it will not find anything malicious. The malicious script is triggered once the user performs a certain action on which the malicious JavaScript loads. It spawns legitimate tools such as PowerShell that run the malicious code directly in the RAM without storing anything on the hard disk. This code triggers the PowerShell to connect to the malicious Command and Control (C&C) server to download and run the final payload directly in the RAM without storing anything on the hard disk [13].

#### Life Cycle of Fileless Malware

**Figure 4:**
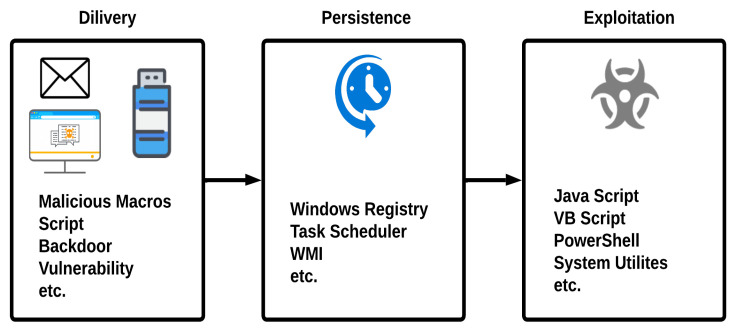
Life cycle of fileless malware.

Figure 4 shows the life cycle of fileless malware. It comprises the following three phases.

- **Delivery phase:** The attacker tries to deliver the malware to the victim’s machine with the help of different techniques mostly used by traditional malware, including social engineering, that encourages the victim to click on the link or send the initial vector of the malware in the email attachment. The attacker’s goal is to deliver the initial payload to the victim machine without triggering the antivirus or anti-malware programs installed on the victim machine. The initial vector may include executables or malicious documents, having malicious macros or scripts embedded in it and delivered through emails, or users downloading them from malicious websites [30].
- **Persistence phase:** Malware uses different evasive techniques to achieve persistence on the victim machine, which means the attack continues even after the system reboots. In the case of fileless malware, the malware resides in the system’s main memory (RAM), which is a volatile memory. The drawback could be that the malicious code could be erased when the system reboots. To overcome this limitation, fileless malware maintains its persistence in the system by making changes to the system registry and by setting the scripts that run automatically after the system reboot, with the help of legitimate tools such as WMI, windows registry, or task scheduler [30].
- **Exploitation phase:** The malware performs the desired action for which it is designed with the help of the legitimate tools installed on the system. If the malware contains scripts or macros, they will be executed and run directly using PowerShell or the command line. Fileless malware uses legitimate tools, such as MS Office, Windows Management Instrumentation (WMI), Windows registry, PowerShell, or task scheduler to perform its operation [30].

### 3.3. File-Based vs. Fileless Malware Comparison

It is worth recalling that fileless malware does not rely on the traditional method of installing itself onto a victim’s computer by creating a new file. Instead, it uses existing tools and processes on the victim’s computer to execute itself and carry out its malicious activities. This can make its detection and prevention more difficult, as it does not leave any visible trace on the victim’s computer.

On the other hand, file-based malware is a type of malware that creates a new file on the victim’s computer as part of its installation process. This file can be detected and removed by antivirus software or any other security tool.

Fileless and file-based malware could be equally dangerous and can cause harm to a victim’s computer or network. For file-based malware detection, there exists a large state-of-the-art, while fileless malware detection is still in its early stages.

Table 2 shows the comparison between file-based and fileless malware features based on their techniques.

## 4. Our Malware Detection Approach

Memory forensics can be an effective way to detect fileless malware. Memory forensics involves analyzing the contents of a computer’s memory (also known as a “memory dump”) in order to identify and extract evidence of malicious activity. By creating a snapshot of the infected machine and a memory dump, one can use a memory forensics tool such as Volatility to extract the fileless malware’s features and train and test a machine learning model. This machine learning model can then be used to detect fileless malware on systems.

**Figure 5:**
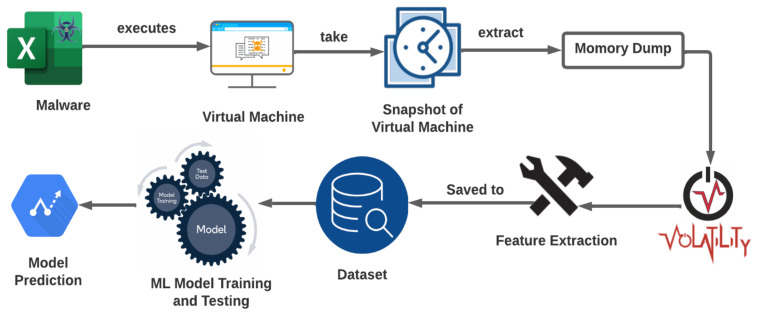
Architecture Of proposed system.

It is important to note that memory forensics is a complex and specialized field, and it requires a thorough understanding of the inner workings of a computer’s memory and operating system. In addition to using a memory forensics tool such as Volatility, it may also be necessary to use other specialized tools and techniques to extract and analyze the relevant information from the memory dump. Overall, the use of memory forensics and machine learning can be a powerful approach for detecting and analyzing fileless malware. Figure 5 shows the architecture diagram of our approach.

The details of our proposed machine learning-based approach toward fileless malware detection are as follows.

### 4.1. Behavior Analysis and Features Extraction

**Figure 6:**
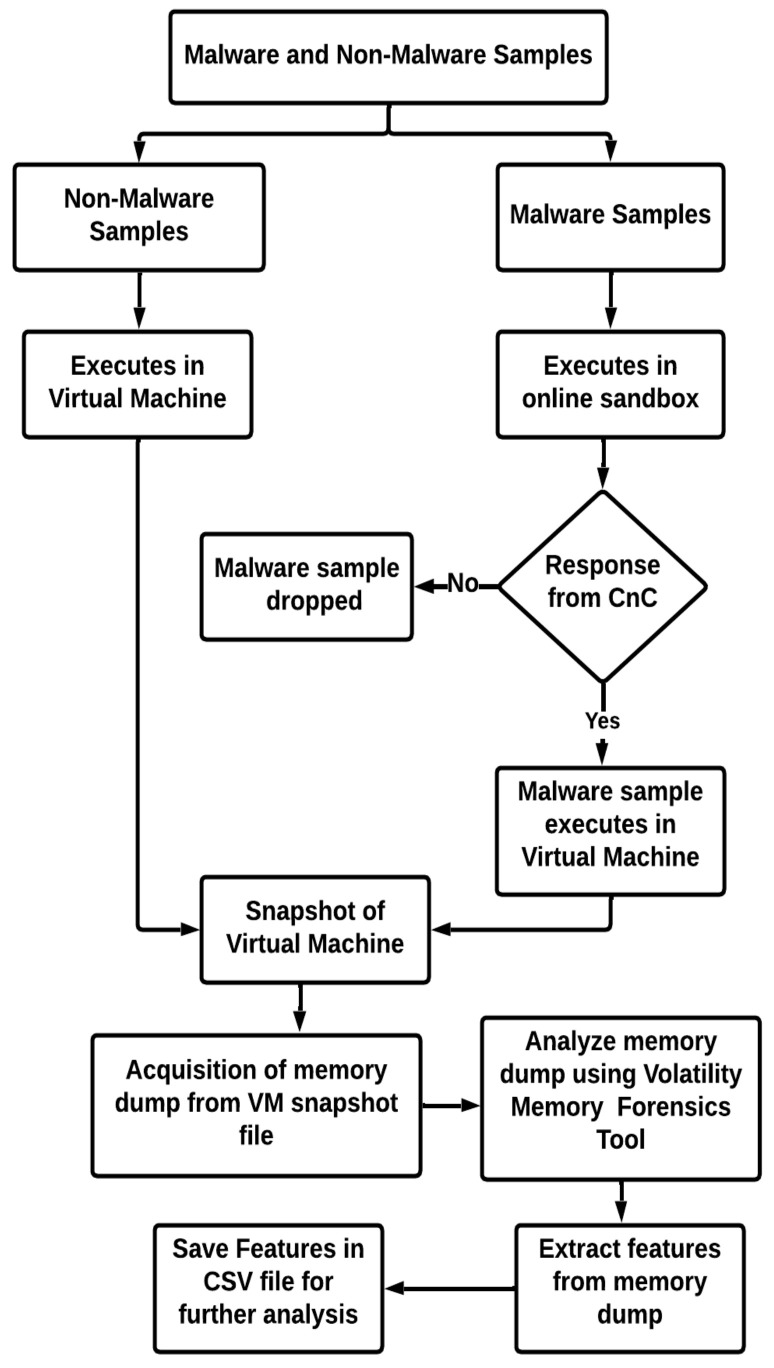
Behavior analysis and features extraction flowchart.

We performed the following steps (as shown in Figure 6) to analyze fileless malware’s behavior and extract its features from the memory dump.

The process starts with the collection of both malware and non-malware samples. The non-malware samples are executed in the virtual machine. In contrast, the malware samples are executed in the online sandbox environment to check whether the Command and Control Server (CnC server) is active by analyzing the network activity. If there is no response from the CnC server against the malware sample, then the malware sample is dropped from further analysis. In case of response, the malware sample is executed in the virtual machine for further analysis. At this point, a snapshot of a virtual machine is taken, which is then used to extract the memory dump of the virtual machine. In the end, the memory dump is analyzed to extract the features. The extracted features are saved to a CSV file and later used as the dataset for training and testing the machine learning model using the Volatility Memory Forensics tool.

### 4.2. Acquisition of Memory Dump from the Virtual Machine

**Figure 7:**
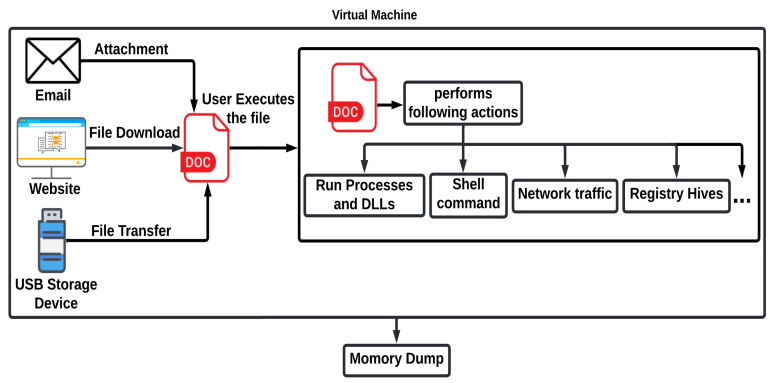
Virtual machine insights.

Fileless malware executes in memory to perform malicious actions, such as creating a new process, using network resources, executing shell commands, making changes in registry hives, etc., as shown in Figure 7. This behavior leads to the use of malware analysis for the detection of fileless malware. In principle, we take the memory dump of the machine and pass it to the volatility memory forensics tool that will help in the analysis of the memory dump. To collect that memory dump, we developed a testbed in a computing environment built using an Intel Core i5-7200U and AMD Radeon R5 M330 platform running Windows 10 Professional. To protect the device from infection, we created a virtual environment with the help of VMWare Workstation 16 by setting up a virtual machine running Windows 7 Service Pack 1. We used a Windows 7-based virtual machine in this study because while analyzing the malware samples, we found that the Volatility memory forensics tool is not working correctly with the memory dumps of the virtual machines running the latest version of Windows. The virtual machine details are shown in Table 3. The VM running Windows OS from the virtualized environment is used to execute the malware and extract the memory dump. To protect the network from infection and to provide network access to the virtual machine, a separate wireless network adapter (TP-Link TL-WN722N) is added to the virtual machine, which is connected to the separate network.

**Figure 8:**
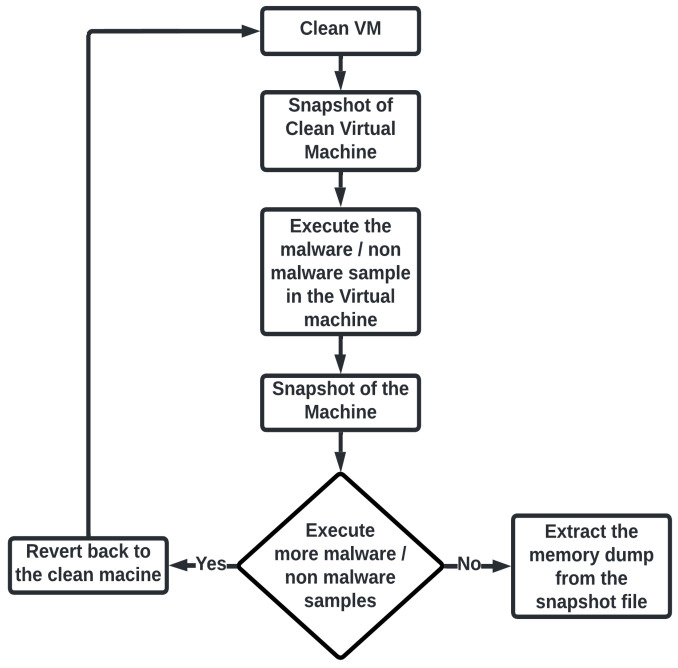
The flow of the snapshot collection process.

VMWare Workstation 16 allows one to take a snapshot of the virtual machines at a specific state and save it to the snapshot file (vmss). This snapshot file extracts the memory dump from the virtual machine. Following is the process to collect the virtual machine snapshots (as shown in Figure 8).

- Step 1—Take a snapshot of the clean virtual machine.
- Step 2—Executes malicious/non-malicious program on the virtual machine.
- Step 3—Take another snapshot of the virtual machine and extract the memory dump.
- Step 4—Revert to the clean instance of the virtual machine, i.e., restore the snapshot of the clean virtual machine.
- Step 5—Extract the memory dump from the snapshot of the virtual machine using VMWare tool-vmss2core.

After collecting the snapshot of the virtual machine, the next step is to extract the memory dump from that snapshot of the virtual machine and to do this VMWare tool vmss2core is used.

### 4.3. Feature Extraction from the Memory Dumps

After acquiring the virtual machine’s memory dump, the next step is to extract the features from that memory dump against the malware or non-malware sample and save them into the CSV file, which will be later used for training and testing the machine learning models. The Volatility Framework tool is used for extracting features from memory dumps. The Volatility tool has over 70 plugins to analyze the different characteristics of main memory. This tool supports 32-bit and 64-bit operating systems, including all Windows, Linux, and macOS flavors. To analyze the memory dump, we first need to initialize the appropriate profile against the memory dump that helps the volatility understand from which operating system the memory dump came. After setting the appropriate profile, we can run different volatility plugins to extract the information, such as a list of the running processes, a list of DLLs loaded by the process, a list of services running, network connections, a list of registry hives, etc. Moreover, as volatility does not give the details of registry events and network information such as DNS requests, etc., the malware sample is also run on the online sandbox AnyRun [31] to extract network and registry-related features. The Volatility plugins used in this study to extract features from the memory dump and the features extracted from the online sandbox are shown in Table 4.

**Figure 9:**
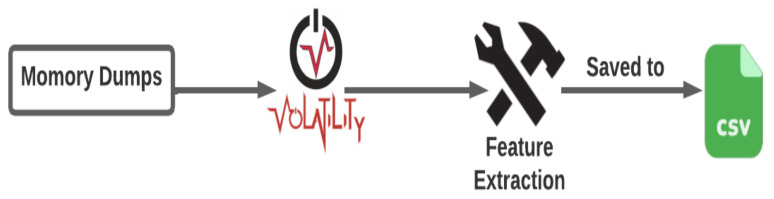
Feature extraction process from memory dump using Volatility.

After extracting the features from the memory dump using these plugins, the extracted features are later used to train and test the machine learning model as shown in Figure 9.

### 4.4. Dataset and Fileless Malware Sample Details

The fileless malware samples are collected from the websites, shown in Table 5. These samples are executed one by one in the virtual machine to extract memory dump from the virtual machine against each malware sample as described in Section 4.2. After acquiring the memory dump, features from each memory dump are extracted with the help of the Volatility Memory Forensics tool as described in Section 4.3. The dataset used in this study is collected from [32], which is an unbalanced dataset because the number of non-malware samples is slightly greater than the number of malware samples in the dataset. We augment this dataset by adding 26 new fileless malware samples to balance the dataset. Among the new fileless malware samples, only five malware samples were executed successfully, and the remaining 21 malware samples did not execute successfully because their command and control server was dead, therefore these 21 fileless malware samples are dropped from the dataset.

### 4.5. Selection of Classifiers

Binary class classification exploits machine learning algorithms to classify data into two categories or classes; in this study, as malware and non-malware (benign). To train and test a classifier, it is usually necessary to split the available data into a training set and a testing set. The training set is used to “train” the classifier by adjusting the model’s parameters to fit the data. In contrast, the testing set is used to evaluate the classifier’s performance on data that it has not seen before. We chose multiple state-of-the-art machine learning algorithms for fileless malware detection, namely, Random Forest (RF), Decision Tree (DT), Support Vector Machine (SVM), Logistic Regression (LR), K-Nearest Neighbor (KNN), XGBoost (XGB), and Gradient Boosting (GB). These are all widely used machine learning algorithms that can effectively classify data into the desired two categories. Each algorithm has its strengths and weaknesses, and which one is best suited for a particular task depends on the characteristics of the data and the requirements of the problem.

## 5. Experimental Evaluation

This section discusses the implementation details and the accuracy of the selected machine-learning algorithms for fileless malware detection.

### 5.1. Experimental Setup for Implementation

For implementing the proposed methodology, the malware samples are analyzed in the virtual environment using VMWare Workstation 16 by setting up a virtual machine running Windows 7 Service Pack 1 equipped with 2 GB of RAM and 40 GB of storage. The machine learning models have been developed and trained on Jupyter notebook, an open-source web-based computing platform for live code execution [35].

Our analysis is based on 45 samples while each of the samples has 33 dimensions. We divide the dataset into train and test splits, where we use 67% of the randomly chosen samples for training the classifiers individually and the remaining 33% samples for testing those pre-trained classifiers. We formulate the problem of fileless malware detection as a binary class classification problem and evaluate all the chosen classifiers (i.e., RF, DT, SVM, LR, KNN, XGB, and GB) on this dataset.

### 5.2. Feature Scaling

It is generally a good idea to scale the features in a dataset before training a machine learning model, especially if the features have very different scales or ranges. This is because many machine learning algorithms use some form of distance measure as part of the learning process, and features with very different scales can dominate the distance measure and make it difficult for the model to learn effectively.

*StandardScaler* (https://scikit-learn.org/stable/modules/generated/sklearn.preprocessing.StandardScaler.html, accessed on 2 November 2022) is a common method for scaling the features of a dataset. It scales the data so that the mean becomes 0 and the standard deviation is 1. This can help the model learn more effectively because the features are on a similar scale, and it can also help prevent some types of numerical instability. Alternately, *MinMaxScaler* (https://scikit-learn.org/stable/modules/generated/sklearn.preprocessing.MinMaxScaler.html, accessed on 2 November 2022) is another method for scaling the features of a dataset. It scales the data so that the minimum value becomes 0 and the maximum value becomes 1. This can be useful for image data, which often has fixed pixel values between 0 and 255 because it ensures that all the features are on the same scale. However, it may not be as appropriate for datasets with a large variance or for features that have a skewed distribution.

In general, it is important to consider the characteristics of the data and the specific requirements of the machine learning algorithm when choosing a method for scaling the features. Both *StandardScaler* and *MinMaxScaler* can be useful in different situations, but it is important to choose the one that is most appropriate for the specific dataset and machine learning problem at hand. To this end, in this paper, we have applied *StandardScaler* for feature scaling.

### 5.3. Parameter Optimization

Parameter optimization is an important step in the process of building and evaluating a classifier, as it can help improve the performance and generalization of the classifier and can also help reduce the computational cost of training and evaluating the classifier. Additionally, it can help prevent overfitting, which occurs when a classifier too closely fits the training data and performs poorly on new, unseen data. Optimizing the parameters allows us to find a balance between fitting the training data well and generalizing it to new data.

It is worth mentioning that we applied 10-fold cross-validation on the train set (split with 67% sample) to find the best hyperparameters and kept the test data unseen by the classifiers to confirm the generalization of the classifiers. Table 6 shows the cross-validation scores of the selected machine-learning algorithms on the train set.

### 5.4. Results

We report the results in terms of True Positive Rate (TPR), False Positive Rate (FPR), and accuracy score. TPR is the fraction of malware samples correctly classified as malware and FPR is the fraction of non-malware samples incorrectly classified as malware and accuracy is the ratio of correct classifications to the overall classification attempts.

**Figure 10:**
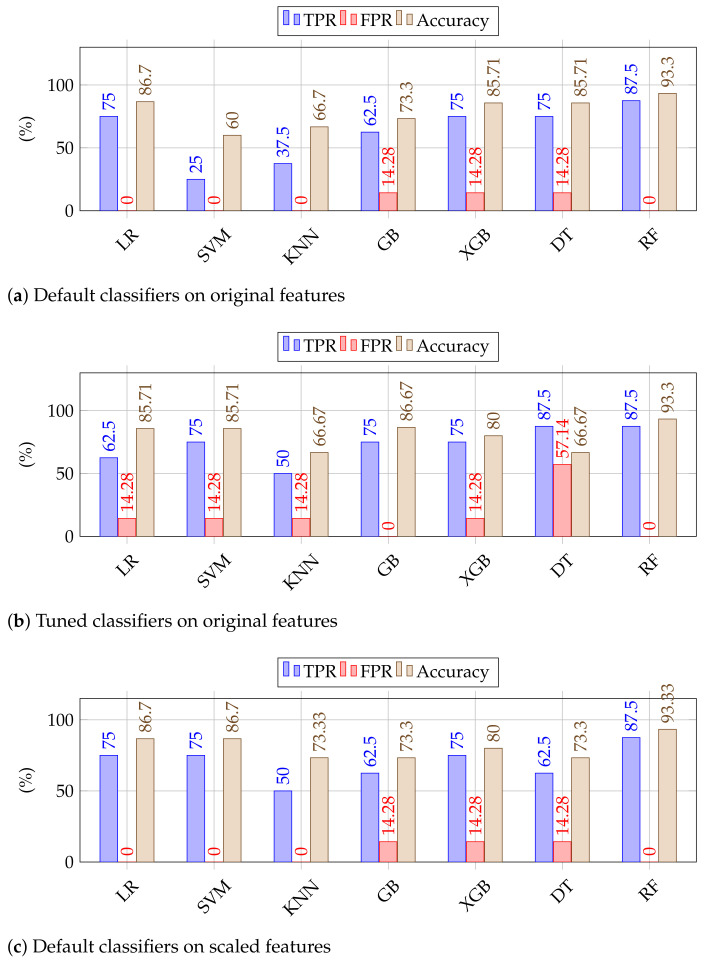

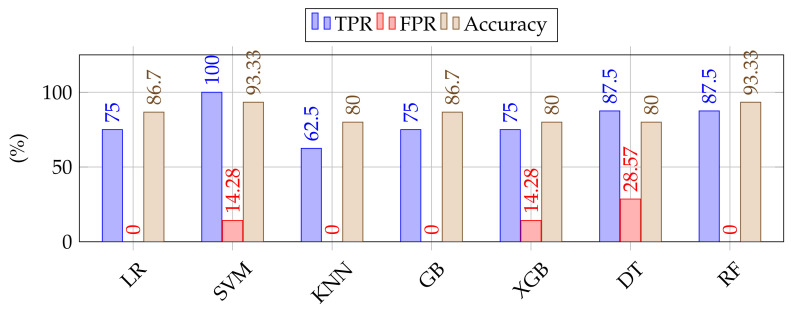
Results of different classifiers (in default settings) on original features (**a**) and scaled features (**c**) and (on best-selected hyperparameters) on original (**b**) and scaled (**d**), respectively.

We summarize our obtained results in Figure 10. In Figure 10a, we show the results of chosen classifiers (with default parameters) on the original features (without scaling). In this setting, we achieved as high as 87.5% TPR at the cost of 0% FPR hence leading to an accuracy of 93.3% by RF classifier. Logistic regression ended up as 2nd best here attaining an overall accuracy of 86.7% (TPR of 75% and FPR of 0). In Figure 10b, we show the obtained results on the same original features but with classifiers trained on optimal parameters. Further in Figure 10c,d, we show the results of classifiers (with default features) and tuned classifiers on scaled data, respectively. As some of the classifiers, e.g., SVM, are not robust enough to high variance and work generally well on scaled data, we see a bit of improvement whereas some classifiers (DT, RF) are extremely robust to these experimental conditions hence they equally perform well scaled/unscaled data. Here we see that RF remained consistent in all experimental settings, and yielded comparatively a higher accuracy of 93.33% (87.5% TPR at the expense of 0% FPR). It is worth mentioning that SVM using optimized parameters, attained 87.5% TPR as RF however it has a higher FPR of 14.28% as compared to 0% for RF.

RF as a classifier has outperformed all other contestants: it remained consistent across all experimental settings which prove its effectiveness against all the odds, i.e., (i) the limited number of samples, (ii) high feature variance, and (iii) without parameter optimization. Hence, it yielded as high as 87.5% TPR at the rate with zero FPR yielding an accuracy of 93.3%. We believe the performance will be improved further if the number of samples is increased.

### 5.5. Discussion

The Random Forest as a classifier works by constructing a collection of decision trees during training and using them to make predictions during testing. At each split in the tree, the algorithm selects a random subset of the features to consider, which helps to decorrelate the trees and reduce overfitting. The final prediction is made by averaging the predictions of all the individual trees. One of the main advantages of RF is that it is relatively easy to use, as it does not require much tuning of hyperparameters [36]. However, it can be computationally expensive to train, especially for large datasets, and it may not perform as well as more complex models on some datasets, which is not the case here. It worked well on our dataset besides the limited number of samples. We believe this classifier will perform well on larger datasets. We consider obtaining an overall accuracy of 93.3% by the RF classifier a good starting point for fileless malware detection. We negate the concern related to its overfitting as this result is obtained on the unseen test dataset, which technically means our classifier already found a good bias–variance tradeoff. Another reason for being so accurate is the absence of noise. If the dataset contains a lot of noise, it may be difficult for the model to learn meaningful patterns and make accurate predictions. A smaller dataset may contain less noise, making it easier for the model to learn and perform better, which is the correct case here.

Fileless malware detection is a relatively newer area; thus, we can only compare our detection accuracy with a similar approach from the literature (i.e., Bucevschi et al. [27]). We managed to obtain 93.3% detection accuracy compared to 83.32%. It is worth noting that Bucevschi et al. did not specify whether the command lines used in their study were from fileless or file-based malware, which could impact the performance of their approach in detecting fileless malware specifically. In addition, it is important to consider the context in which the approaches were evaluated, including the dataset(s) used to evaluate the performance of the models and any other relevant factors that could impact the performance of the approaches.

It is not uncommon for RF to perform well on various tasks, especially when the data are noisy or have a high degree of variance. However, it is always important to evaluate the performance of any machine learning model in the context of the specific problem being solved and to compare it to other possible approaches to see which one performs best. It is also important to keep in mind that increasing the number of samples may improve performance, depending on the specific characteristics of the data.

### 5.6. Limitations

It is common for datasets in the field of cybersecurity to be limited in size, as it is difficult to obtain large numbers of malicious samples. This makes it challenging to train and evaluate machine learning models, as the models do not have sufficient training samples to learn from. In the case of fileless malware, it can be particularly difficult to obtain samples because the CnC servers may no longer be active, or the samples may only work under certain conditions. Excluding these samples from your analysis is reasonable, as they may not represent the types of fileless malware you are interested in detecting. One way to potentially overcome the limited size of the dataset is to use data augmentation techniques to generate additional synthetic samples. This can help increase the dataset’s size and improve the model’s performance. We leave to use these augmentation and fine-tuning techniques as related work.

## 6. Conclusions and Future Work

Fileless malware is a type of malware that does not rely on installing itself as a file on the infected system’s hard drive. Instead, it runs directly in the main memory (RAM) and leverages legitimate programs or operating system tools to carry out its malicious activities. This makes it difficult to detect because it does not leave behind the usual traces of malware, such as files or registry entries. The life cycle of fileless malware typically begins with the initial infection, which may occur through various means, such as phishing attacks, drive-by downloads, or exploitation of vulnerabilities in software. The malware then establishes itself in the main memory and begins to carry out its malicious activities, such as stealing sensitive data, installing additional malware, or taking control of the infected system.

To detect fileless malware, it is important to focus on identifying the presence of malicious activity rather than the presence of malware files. One approach to detecting fileless malware is to analyze the features of the system’s main memory, looking for indicators of malicious activity such as changes to system files or the use of unusual network communication patterns. Machine learning techniques can be used to analyze these features and identify patterns indicative of fileless malware. We have also exploited a machine learning approach to develop our fileless malware detection model. By training the model on a dataset of fileless malware and benign samples and then evaluating the model’s performance on a testing dataset, we could select the best-performing model and tune its hyperparameters to improve its accuracy. It is worth mentioning that Random Forest achieved the highest accuracy of 93.3% with a TPR of 87.5% at an FPR of 0% on the unseen test set. The metrics illustrate the models’ ability to correctly identify fileless malware and non-fileless malware (benign) samples with high precision.

We plan to improve the efficiency of our proposed fileless malware detection method. One potential direction for future work could be incorporating features related to process creation and network connections into the machine-learning model. The model can distinguish malicious and legitimate processes more accurately by considering a broader range of features. Another potential direction for improvement could be to incorporate additional machine learning techniques, such as deep learning approaches, which have shown to be effective in a variety of tasks, including malware detection. Deep learning approaches can learn complex patterns in data and can be particularly effective when working with large, high-dimensional datasets. It may also be useful to consider incorporating other types of features, such as those related to system behavior, system configurations, and user activity, which can provide additional context for the machine learning model to consider when making predictions. Finally, it may be beneficial to conduct further evaluations of the model’s performance on a diverse and representative dataset to ensure that the model is robust and can generalize to new situations.

## Figures and Tables

**Table 1:** Comparison of the proposed approach with state-of-the-art approaches.

	Type of Malware	Vulnerabilities Exploitation	Analysis Technique	Dataset Used	Classification	Detection
**[10]**	In memory attack	Javascript and HTML5	Tested on anti-malware detection tools	NA	No	No
**[8]**	In memory and registry attacks, non portable executable	Macros, scripts, registry values, Powershell, SMB payload, obfuscated code, DLL files etc.	Analyzed published and Kuckoo Sandbox samples attack mapped with MITRE	Collected samples from hybrid analysis and GitHub	Yes	No
**[21]**	In-memory attack	Shell commands, file system, data-flow, SSH server in IoT	By Profiling four s/w and h/w honeypots in multiple public cloud	NA	No	No
**[24]**	In-browser cryptojacking attacks	PowerShell, WMI, scheduler, and registry	Patterns of Fileless malware for Tactics, Techniques, and Procedures	NA	Yes	No
**[9]**	In memory and browser-based attacks	Shell commands, file system, SMB, macro, browsers	NA	NA	Yes	No
**[11]**	In system and in network attacks	Windows Registry Task scheduler WMI etc.,	Runtime behavior of the system	NA	No	No
**[16]**	In sensors memory	IoT devices and edge computing devices	ML-based model with sigmoid function	NA	Yes	Yes
**[28]**	In-Memory attacks	Windows PowerShell scripts	Analysis of registry, commands and windows security logs, abnormal processes	Own Dataset (RAM dump)	Yes	No
**[27]**	In-Memory attacks	Command lines, PowerShell, WMI and bash scripts	Observation of Malicious commands, anomalies, and obfuscation methods	Built own dataset 500,551 command line, PowerShell and WMI Scripts, etc.	No	Yes
**Our Approach**	In-Memory and browser attacks with 33 different features	PowerShell, WMI, Macros, VB script	Machine Learning and Memory Forensics	Virus Total, AnyRun, and PloySwarm	Yes	Yes

**Table 2:** Comparison between file-based and fileless malware.

Features	File-Based Malware	Fileless Malware
Source Code, Malicious File	Available	Not Available
Detection Complexity	Moderate	High
Persistence	Medium	Low
File Types	Executables	JavaScript, VBScript, Macros, PowerShell, WMI
Target	Single OS	Can target multiple OS
Obfuscation Techniques	File encryption, code embedding, etc.	Encoding, Encryption etc.

**Table 3:** Detail of Virtualized Environment.

OS	RAM	Storage	Network
Windows 7 SP 1	2 GB	40 GB	Host Only with separate network adapter

**Table 4:** List of Volatility plugins used to extract features from memory dumps and the features extracted from the online sandbox.

Name	Description	Type
pslist	Display the list of running processes	Plugin
pstree	Display the list of the relationship of parent-child processes	Plugin
preview	Display the list of hidden processes	Plugin
dlllist	Display the list of Dlls used by each process	Plugin
mutantscan	Display the list of windows thread based mutexes	Plugin
svcscan	Display the list of windows services	Plugin
handles	Display the list of open handles of each process	Plugin
netscan	Scan and Display the list of TCP connections and sockets	Plugin
ldrmodules	Display the list of unlinked Dll’s	Plugin
modules	Display the list of loaded modules	Plugin
privs	Display the list of processes having privileges	Plugin
callbacks	Display the list of kernel routines	Plugin
hivelist	Display the list of registry hives	Plugin
thrdscan	Find and display the list of thread objects present in the main memory	Plugin
total registry events	Display the total number of registry events	Sandbox
registry read events	Display the number of registry read events	Sandbox
registry write events	Display the number of registry write events	Sandbox
registry delete events	Display the number of registry delete events	Sandbox
number of exe files dropped	Display the number of exe files dropped	Sandbox
number of files with unknown types dropped	Display the number of files with unknown file types dropped	Sandbox
number of HTTP, HTTPS requests	Display the number of HTTP, HTTPS requests	Sandbox
number of DNS requests	Display the number of DNS requests	Sandbox

**Table 5:** Fileless malware samples collected.

SR.	Name of Website	Description	Published Year	Owned By	No. of Malware Samples Available	No. of Fileless Malware Samples Available and Downloaded
1	VirusShare [33]	A repository of live malware samples with malicious code	2011	Corvus Forensics	55,372,441	11
2	AnyRun [31]	An online interactive sandbox with a vast malware sample database	2016	AnyRun	6,200,000	10
3	PolySwarm [34]	A crowdsourced threat detection marketplace	2018	PolySwarm	350,000	5

**Table 6:** Computed best parameters with obtained CV score for different classifiers.

Classifier	Parameters	Score
Logistic Regression	class_weight = “balanced”, fit_intercept = True, solver = “liblinear”	77.0
Support Vector Machine	C = 50, gamma = “scale”, kernel = “rbf”, class_weight = “balanced”, degree = 3, shrinking = False	76.5
Gradient Boosting	max_depth = 1, n_estimators = 100	90.0
KNearestNeighbor	n_neighbors = 3, weights = “uniform”, algorithm = “auto”, leaf_size = 5	60.5
XGB	gamma = 0.05, max_depth = 3, min_child_weight = 1, n_estimators = 10, reg_alpha = 0.5, reg_lambda = 1.0, subsample = 0.8	93.0
Decision Tree	class_weight = “balanced”, criterion = “gini”, max_features = “sqrt”, min_samples_split = 3, splitter = “best”	90.9
Random Forest	bootstrap = False, criterion = “gini”, n_estimators = 50, max_features = “sqrt”	97.5

## Data Availability

Available upon request.

## Abbreviations

The following abbreviations are used in this manuscript:

CnC	Command and Control Server
RAM	Random Access Memory
WMI	Windows Management Instrumentation
DLL	Dynamic-link Library
SSH	Secure Shell
IoT	Internet of Things
CSV	Comma Separated Values
VM	Virtual Machine
